# The Pellicle–Another Strategy of the Root Apex Protection against Mechanical Stress?

**DOI:** 10.3390/ijms222312711

**Published:** 2021-11-24

**Authors:** Izabela Potocka, Joanna Szymanowska-Pułka

**Affiliations:** Faculty of Natural Sciences, Institute of Biology, Biotechnology and Environmental Protection, University of Silesia in Katowice, Jagiellońska 28, 40-032 Katowice, Poland

**Keywords:** pellicle, mechanical stress, root apex, epidermal surface, confocal laser scanning microscopy, scanning electron microscopy

## Abstract

In grasses, the apical part of the root is covered by a two-layered deposit of extracellular material, the pellicle, which together with the outer periclinal wall of protodermal cells forms the three-layered epidermal surface. In this study, the effect of mechanical stress on the pellicle was examined. An experiment was performed, in which maize roots were grown in narrow diameter plastic tubes with conical endings for 24 h. Two groups of experimental roots were included in the analysis: stressed (S) roots, whose tips did not grow out of the tubes, and recovering (R) roots, whose apices grew out of the tube. Control (C) roots grew freely between the layers of moist filter paper. Scanning electron microscopy and confocal microscopy analysis revealed microdamage in all the layers of the epidermal surface of S roots, however, protodermal cells in the meristematic zone remained viable. The outermost pellicle layer was twice as thick as in C roots. In R roots, large areas of dead cells were observed between the meristematic zone and the transition zone. The pellicle was defective with a discontinuous and irregular outermost layer. In the meristematic zone the pellicle was undamaged and the protodermal cells were intact. The results lead to the conclusion that the pellicle may prevent damage to protodermal cells, thus protecting the root apical meristem from the negative effects of mechano-stress.

## 1. Introduction

In field conditions roots penetrate and explore soil in order to grow and to acquire water and nutrients. If the soil particles are small and easily movable penetration is undisturbed and the root tip grows in a predictable way, that is vertically, with a more or less steady rate. However, in medium in which obstacles are present, such as large unmovable particles, fixed objects, or rigid thin pores, the trajectory of the root and its growth rate undergo modification [1,2]. Independent of the type of the medium, a penetrating root senses mechanical force exerted by soil particles and/or obstacles.

**Figure 1:**
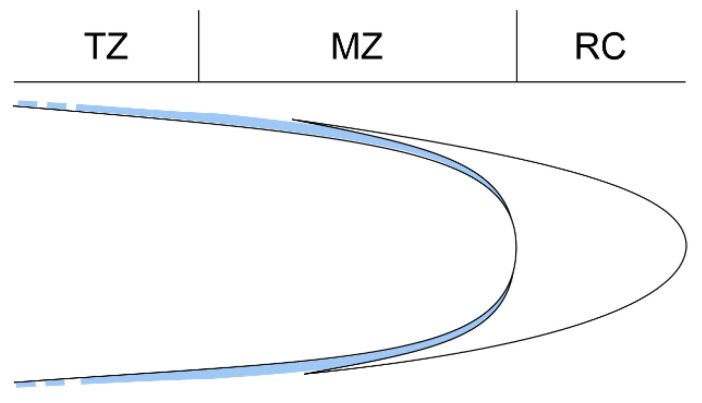
Localization of the pellicle (light blue) along the root proper, the pellicle becomes thinner and gradually disintegrates (dashed line) above the transition zone. RC root cap, MZ meristematic zone, TZ transition zone.

The first and most direct area of interaction between root and soil is at the root’s outermost cell layer epidermis. In the root apex the epidermis is exposed not only to contact with the medium, but also to mechanical forces resulting from growth and friction. In grasses, protodermal cells of the root apex are covered by the two-layered deposit of extracellular material named the pellicle (Figure 1) [3]. This product of protodermal cells together with an outer periclinal cell wall form the so-called epidermal surface whose function is stiffening the root tip and cementing cell files of young epidermis [4,5]. Properties of the epidermal surface are easily observable because it can be removed intact from the root tip [6]. Subsequent layers of the epidermal surface are referred to as L1, L2, and L3, which corresponds to the primary cell wall, the inner thick pellicle layer and the outer thin pellicle layer, respectively. These layers are clearly recognizable based on specific staining properties and on characteristic structural features. The epidermal surface as a whole is polysaccharide in nature and strongly reacts to periodic acid–Schiff (PAS) staining [7]. When toluidine blue (specific to acidic polysaccharides) is applied, a staining is observed in L1 and L3, while L2 remains mostly unstained [7]. In addition, in response to Calcofluor White, L1 and L3 exhibit a more intense fluorescence signal than the middle layer, L2 [7,8]. The outer epidermal wall (L1) and both pellicle layers (L2, L3) show a different fibrillar arrangement. The L1 is polylamellar with a helicoidal pattern of microfibrils, while in the L2, closely packed fibrils are oriented parallel to the root axis. In the L3, fibrils are coarser and form a less regular mesh [3,4]. The thickness of the pellicle layers changes along the root axis: they are thickest in the meristematic zone; further, the L3 undergoes disintegration and is lost in the elongation zone, while the L2 becomes thinner and thinner and eventually disintegrates and takes the form of single patches at the base of root hairs [3].

External mechanical stress applied to the root apex results in serious modifications in root morphology and anatomy (see [9]). The most obvious changes are swelling and shortening of the root [10,11], often associated with an increased diameter of the stele [12] or modified cell morphology, such as increased radial dimension and decreased cell length of cortical cells [13,14,15]. In some experiments with roots exposed to mechanical stress, anatomical alterations in vascular tissue [16], atypical orientation of cell divisions [17] and modification of the root meristem organization [17,18] have also been observed. Unfortunately, little is known about the response of the root epidermis to mechanical impedance. The only reported results refer to the altered number and size or to deformation of mature epidermal cells in some cereals [12,19], in lupine [15] and in *Arabidopsis* [20]. However, no data are known about a potential effect of mechanical force on the epidermal surface in the meristematic zone of the root apex.

As a research object, the epidermal surface, and in particular the pellicle, is a bit forgotten nowadays. Its function and structure have not been studied since the above-cited excellent work by McCully and co-workers [3,4,5,6,7,8]. The pellicle was also mentioned over 20 years ago in a paper presenting methods for detecting arabinogalactan proteins on the plant surface [21]. Moreover, the nature of the root epidermal surface seems to be poorly recognized as most researchers have mistakenly interpreted the surface layers as root mucilage, as an element of the complex epidermal cell wall or even as an air space between the epidermis and root cap [22,23,24,25]. Also unknown is the response of the pellicle to external stress, particularly mechanical stress, which is commonly found in soil, the natural growth environment of the root.

In the current study, we investigate if and how the epidermal surface reacts to externally applied mechanical stress. For this purpose, we used the previously described experimental design [18] in which maize roots were grown in a tiny, rigid pore that resulted in dramatic changes both in the form and cell pattern of its apex. However, in the current study we do not focus on morphology but on the potential ability of the pellicle to protect the meristematic zone of the root. This work is also an attempt to add to our knowledge in the field through in-depth research on the microstructure of the epidermal surface with the use of three-dimensional (3D) imaging techniques: confocal laser scanning microscopy (CLSM) and scanning electron microscopy (SEM).

## 2. Materials and Methods

### 2.1. Plant Material

Maize seeds (*Zea mays* L. subsp. *mays*, *saccharate* group, cv. Złota Karłowa) were sterilized with 10% commercial bleach solution for 15 min and washed thoroughly with running tap water. The seeds were germinated between two layers of wet filter paper, which were rolled and inserted in a glass beaker partly filled with deionized water and covered with aluminum foil. The beakers were kept in darkness at 22 °C for 3–4 days. For the experiment, only the seedlings with 15–20 mm long straight primary roots were used. The average root diameter in its cylindrical part was about 1 mm.

### 2.2. Experiment

**Figure 2:**
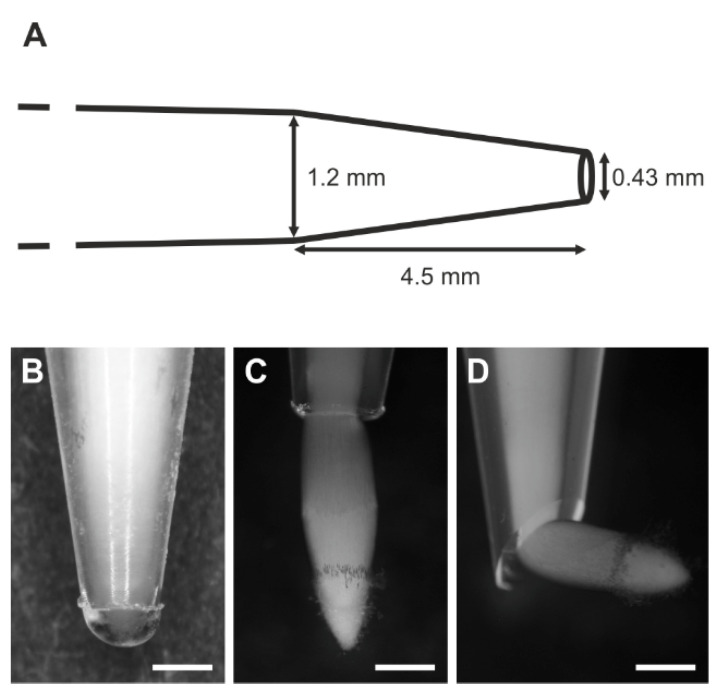
The experimental set-up. (**A**) Geometrical dimensions of the plastic tube. (**B**–**D**) The analyzed stages/variants of the experiment: stressed root apex reaching the tube ending (**B**), recovering root apex growing out of the tube in untouched (**C**) and obliquely cut (**D**) tube ending. Scale bars: 0.5 mm.

The selected roots (ten roots in each of thirty repetitions) were introduced into plastic tubes whose endings were conical in shape. The internal diameter of the tube in its upper cylindrical part was approximately 1.2 mm while the internal diameter of the tube tip was approximately 0.43 mm (Figure 2A). The tubes were fixed vertically in a glass container and kept in a moist environment (85% relative humidity) at 22 °C. The roots grew freely until they reached the region where the tube diameter became smaller than the diameter of the root and where the root apices encountered mechanical impedance (see also [18]). After 24 h, the roots whose apices reached or were about to reach the ending of the tube (stressed roots S, Figure 2B) and the roots that grew out of the tube (recovering roots R, Figure 2C) were taken for microscopic analyses. Roots growing freely between the layers of moist filter paper served as controls (C). Fifty to sixty roots of each of the three groups (C, S, R) were used. To verify a possible influence of the stress distribution on the root growth some roots were introduced in tubes whose endings were obliquely cut (Figure 2D). In this variant, only the R roots were analyzed.

For microscopic examination, the stressed roots (S) were carefully removed from the tubes and treated according to the applied procedure (see below), while recovering roots (R) were not removed in order not to damage them, so they were further treated fixed in the tube.

### 2.3. Cell Viability Assays

#### 2.3.1. Evans Blue Staining

Whole roots (fifteen to twenty roots of each of the three groups C, S, R) were incubated in 0.25% (*w*/*v*) aqueous solution of Evans Blue(Sigma, E2129, Darmstadt, Germany) for 15 min at room temperature, then washed thoroughly several times with deionized water and immediately photographed with a Nikon SMZ800 stereomicroscope and a Nikon DS-Fi1 camera. The stained apices were free-hand cut to approximately 100 μm thick cross sections at the basal limit of the meristem, that is about 1 mm from the root/cap junction and at the middle of the transition zone (TZ), that is about 2 mm from the root/cap junction [26,27]. In recovering apices (R), the Evans Blue dye indicated two regions of different shades, so these apices were additionally cut on the boundary between the regions, that is between 1 and 2 mm from the root/cap junction. Ten to fifteen sections taken from each of the above-mentioned levels of the three groups of roots (C, S, R) were examined. To have a closer look at the two differently stained regions occurring in R apices, their longitudinal tangential sections were taken. The sections were mounted in water and observed with a Nikon Eclipse 80i microscope using bright field and Nomarski optics, then photographed with a Nikon DS-Fi2 camera.

#### 2.3.2. Propidium Iodide (PI) Staining

Apical parts of the roots, 0.5 mm long (in R roots, a fragment protruding from the tube), were cut off, stained with 10 μg/mL propidium iodide in deionized water (Sigma, P4170, Darmstadt, Germany) for 15 min and rinsed twice in deionized water. Next, the samples were placed in a water chamber between two coverslips separated by a silicone spacer frame (cut from a Press-to-Seal silicone sheet with adhesive, ThermoFisher Scientific, Waltham, MA, USA) to prevent sample crushing. Images were acquired in a plane tangential to the root surface using an Olympus FluoView FV1000 confocal laser scanning microscope with a UPlanFL N 20× objective (NA 0.50). PI was excited at 543 nm with a helium-neon laser and emission was collected at 575–675 nm. Stacks of optical sections (image size 1024 × 1024 pixels, pixel size 0.207 μm) were taken with an interval of 1 μm. Image processing was performed applying ImageJ software. Ten apices from each of the three groups (C, S, R) were analyzed.

### 2.4. Visualization of the Epidermal Surface Components

#### 2.4.1. Coomassie Blue Staining

To visualize the outer pellicle layer, L3, whole roots (fifteen to twenty roots of each of the three groups C, S, R) were stained with 0.05% (*w*/*v*) aqueous solution of Coomassie brilliant blue R-250 (Pol-Aura, PA-03-3543-T, Dywity, Poland) for 15 min at room temperature. Next, the apices were washed with deionized water, hand cut as described above for the Evans Blue procedure and photographed with a Nikon Eclipse 80i microscope and a Nikon DS-Fi2 camera.

#### 2.4.2. Confocal Laser Scanning Microscopy (CLSM) Imaging

**Figure 3:**
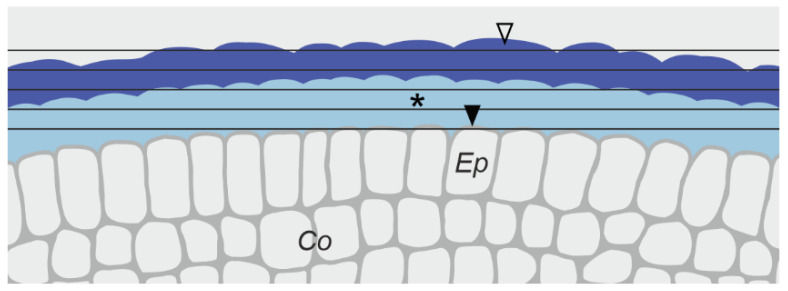
Schematic representation of the epidermal surface imaging with the use of the confocal laser scanning microscope. Black straight lines indicate the optical planes at which fluorescence was captured. The individual layers of the epidermal surface, the L1, L2 and L3, are demarcated by a solid arrowhead, asterisk and empty arrowhead, respectively. Ep immature epidermis, Co cortex.

Apical portions (approximately 1 cm) of C and S roots (ten roots of each of the two groups) were stained with 0.01% Calcofluor White M2R (Sigma, F3543, Darmstadt, Germany) in phosphate-buffered saline (PBS, pH 7.2) for 15 min in darkness and washed three times with deionized water. Next, the roots were split centrally along their main axes and the halves were placed in a water chamber between two coverslips separated by a silicone spacer frame (Press-to-Seal silicone sheet with adhesive, ThermoFisher Scientific). Images of the two layers of pellicle (L3, L2) and of the outer protodermal cell wall (L1) were acquired in a plane tangential to the root surface (see Figure 3) using an Olympus FluoView FV1000 confocal laser scanning microscope with a UPlanSApo 60× oil immersion objective (NA 1.35). Calcofluor White was excited with a 405 nm laser diode and emission was collected between 425 and 525 nm. Z-stacks of optical sections (image size 1024 × 1024 pixels, pixel size 0.103 μm) were taken with an interval of 0.13 μm. Image processing, including orthogonal projections and 3D reconstructions of z-stacks, was performed applying ImageJ software.

Application details of the used staining techniques are specified in Table A1 (Appendix B).

#### 2.4.3. Scanning Electron Microscopy (SEM) Imaging

Apical parts of roots (in R roots, a fragment protruding from the tube) were cut off and immediately fixed in 100% methanol for 1 h at room temperature [28]. Next, the samples were dehydrated in 100% ethanol for 30 min, transferred to fresh 100% ethanol and left overnight. After dehydration, a critical point drying procedure with the EM CPD300 Automated Critical Point Dryer (Leica Microsystems, Wetzlar, Germany) was done following the ‘Rice Root’ protocol according to the manufacturer’s instructions. Dried apices were mounted on aluminum stubs with double sided adhesive carbon tabs and sputter coated with a 10–15 nm thick gold film in a Pelco SC-6 sputter coater (Ted Pella, Inc., Redding, CA, USA). The specimens were observed using a UHR FE-SEM Hitachi SU8010 field emission scanning electron microscope (Hitachi High-Technologies Corporation, Tokyo, Japan) at accelerating voltages of 7 kV and 10 kV and a working distance of 8–10 mm. In C and S roots, micrographs were collected in the meristematic zone (about 0.5–1 mm from the root tip). In case of R roots, two regions were imaged: below and above the boundary between two shades of Evans Blue stain. Five to seven apices from each of the three groups (C, S, R) were analyzed.

## 3. Results

### 3.1. Root Growth Orientation

The orientation of all the control (C) roots grown between the layers of moist filter paper was vertical. Both in undisturbed tubes and in tubes with obliquely cut endings, the stressed (S) roots grew in a similar way encountering increasing pressure from the tube, due to its decreasing diameter (Figure 2B). In undisturbed tubes the recovering (R) roots maintained a vertical orientation (Figure 2C), while in tubes with obliquely cut endings they changed their orientation to horizontal immediately after leaving the tube (Figure 2D).

### 3.2. Viability of the Root Cells

**Figure 4:**
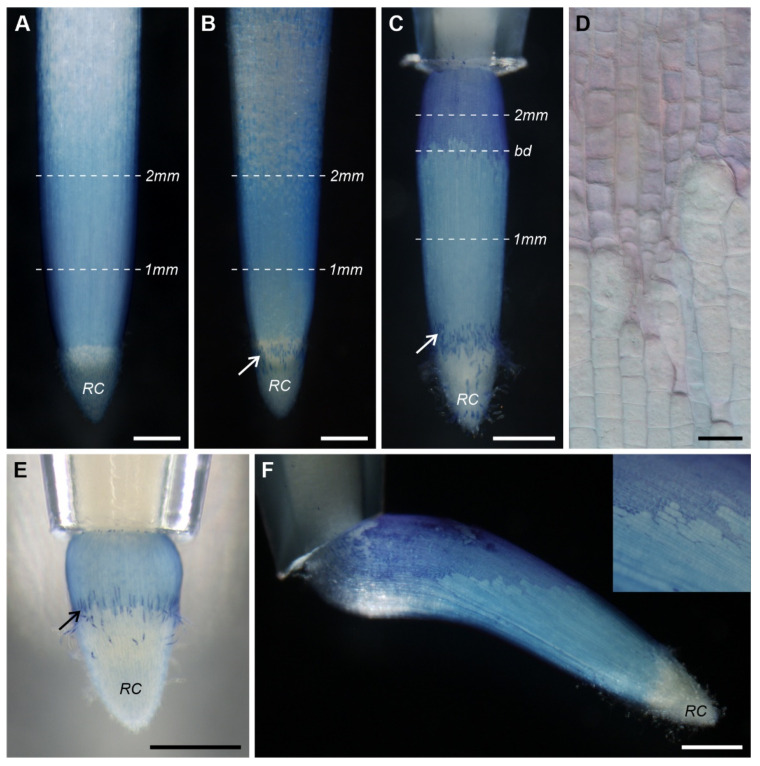
Overall view of the C, S and R root tips dyed with Evans Blue (**A**–**C**, respectively). Dashed lines indicate the level of cross sections shown in Figures 5 and 7, see Materials and Methods. (**D**) Magnified view of the R root surface showing the irregular character of the boundary (bd) separating two regions of different shades of stain. (**E**) Evans Blue-stained recovering root whose apex has just left the tube ending; notice the lack of an area of dark blue staining. (**F**) Evans Blue-stained recovering root growing out of the tube with obliquely cut ending and magnified view of the root surface (upper right corner). Arrows in (**B**,**C**,**E**) point to dead cells of the root cap (RC). Scale bars: 0.5 mm (**A**–**C**,**E**,**F**), 20 μm (**D**).

Evans Blue staining was observed on the surface of roots of all groups (C, S, R). In the C and S apices the stain was more or less continuous (Figure 4A,B) and reached up to 3 mm from the root tip, while in the R apices a clear boundary separating two regions of different shades of stain occurred: in the lower region (slightly above the root cap) the staining was pale blue and in the upper region it was dark blue (Figure 4C). The boundary was not smooth, but appeared perpendicular to the root axis, although it ran at different levels in separate files of protodermal cells (Figure 4D). Moreover, in roots that had just exited the tube only pale blue stain was visible (Figure 4E). In horizontally growing roots in the tubes with obliquely cut endings, the boundary became more or less parallel in reference to the root axis, yet it was still not smooth (Figure 4F). Root caps remained unstained; only single, dark blue patches were visible (Figure 4B,C,E).

**Figure 5:**
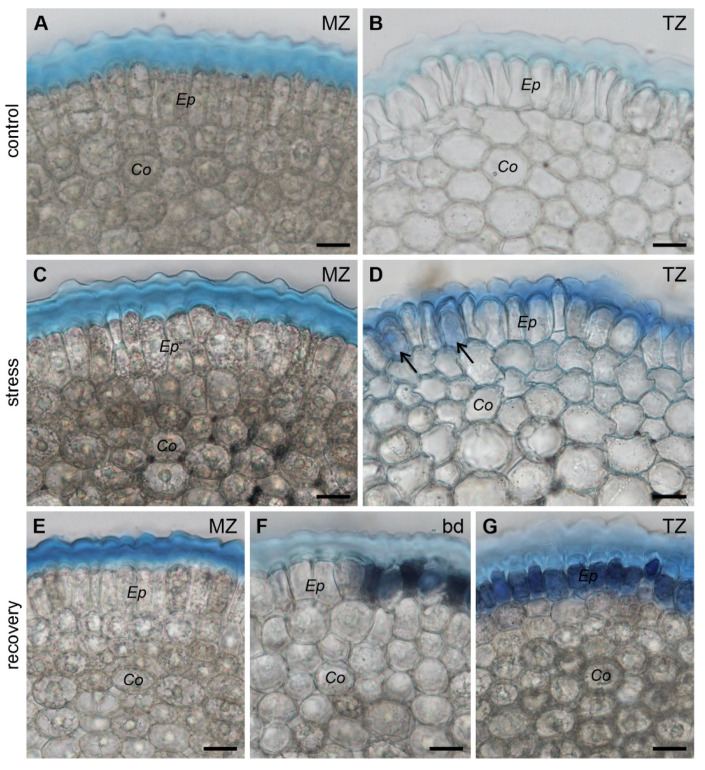
Cross sections of the roots stained with Evans Blue. Cut at the level of 1 mm from the root/cap junction (meristematic zone) in C, S and R roots (**A**,**C**,**E**, respectively), at the level of 2 mm from the root/cap junction (transition zone, **B**,**D**,**G**, respectively) and at the boundary between differentially stained regions in R roots (**F**). Arrows in (**D**) indicate dead epidermal cells. Ep immature epidermis, Co cortex, MZ meristematic zone, TZ transition zone, bd boundary between differentially stained regions in R roots. Scale bars: 20 μm.

Since Evans Blue typically stains dead or damaged cells with ruptured or destabilized cell membranes, we checked cell viability on cross sections of the apices at various levels (white dashed lines in Figure 4A–C). In control roots young epidermal and cortical cells appeared unstained (live) at both analyzed levels (Figure 5A,B). In the stressed roots, only cells in the meristematic zone (1 mm from the root/cap junction) were completely free from the dye (Figure 5C), while in the transition zone (2 mm from the root/cap junction), single dead (dark blue) cells could be observed in the epidermis (Figure 5D). In recovering roots, the cells below the boundary between the regions of different shades of dye (meristematic zone) were living (unstained, Figure 5E), whereas at the boundary, some of the cells were alive and some were dead due to the irregular character of the boundary (Figure 5F). Eventually, in the region above the boundary (transition zone) all the young epidermal cells were dead (dark blue), while the cortical cells remained unstained (Figure 5G). Further observations of the R roots revealed that despite the epidermal cell death, growth of these roots was not inhibited (not shown), although in some cases a temporary reduction in growth was observed.

**Figure 6:**
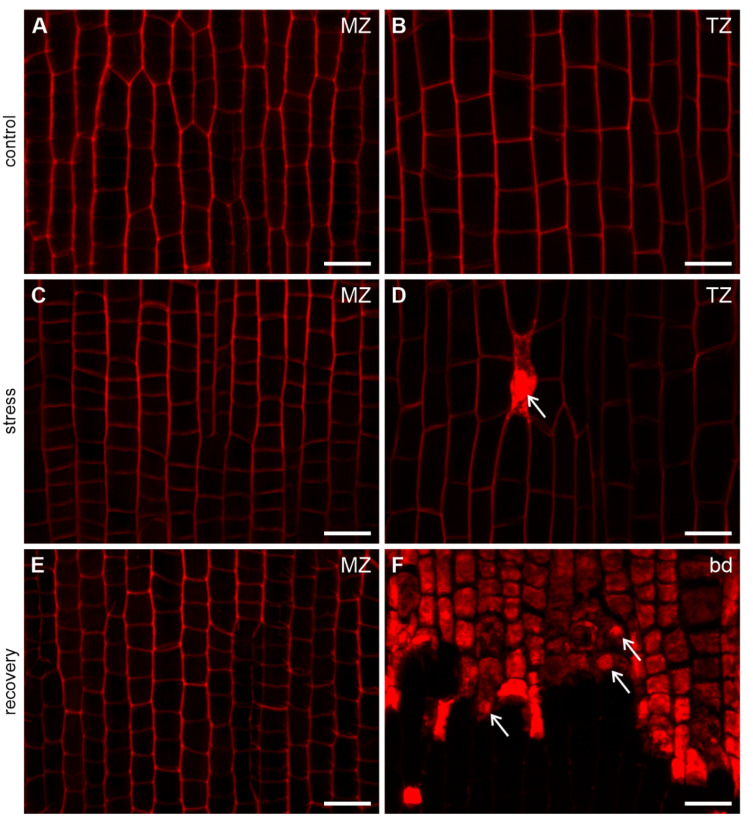
Viability detection of protodermal (**A**,**C**,**E**) and young epidermal (**B**,**D**,**F**) cells with propidium iodide. In the meristematic zone of C, S and R roots (**A**,**C**,**E**, respectively) and in the transition zone of C roots (**B**) all cells are viable (only cell walls stained). In the transition zone of S roots (**D**) rarely distributed single cells are dead (cytoplasm and nuclei stained, arrow). In the boundary region of R roots (**F**) all cells above the boundary are dead with cytoplasm and nuclei (arrows) stained, while cells below the boundary are alive, although their walls are not visible due to reduced laser power to avoid oversaturated pixels resulting from the very strong signal in cells above the boundary. MZ meristematic zone, TZ transition zone, bd boundary between differentially stained regions in R roots. Scale bars: 20 μm.

Cell viability was also determined via propidium iodide staining. All young epidermal cells of control roots in both the meristematic zone and the transition zone were viable, having only cell walls stained (Figure 6A,B). The same staining pattern was observed in the meristematic zone of the S roots (Figure 6C), while in the transition zone of these roots, single dead epidermal cells showed bright red fluorescence in nuclei (Figure 6D). In R roots, all protodermal cells of the meristematic zone were viable (Figure 6E). Between the meristematic and transition zones, the staining pattern was similar to that of Evans Blue staining, that is, a clear boundary separating the regions of viable cells and non-viable cells with heavily stained cytoplasm and nuclei was visible (Figure 6F). As in the Evans Blue-dyed roots, this boundary was uneven and it ran at different levels in particular files of protodermal cells.

### 3.3. Changes in the Structure of the Epidermal Surface under Mechanical Stress

#### 3.3.1. Changes in the Pellicle Layers

**Figure 7:**
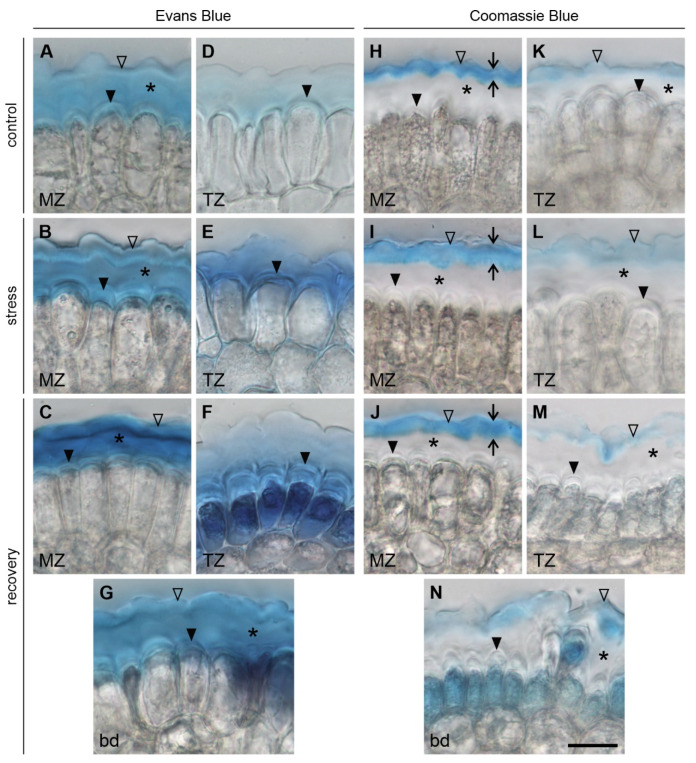
Cross sections of the roots stained with Evans Blue (**A**–**G**) and Coomassie blue (**H**–**N**). Cut at the level of 1 mm from the root/cap junction (meristematic zone) in C, S and R roots (**A**–**C**,**H**–**J**), at the level of 2 mm from the root/cap junction (transition zone, **D**–**F**,**K**–**M**) and at the boundary between differentially stained regions in R roots (**G**,**N**). Short arrows in (**H**–**J**) show the L3 thickness. Solid arrowheads, asterisks and empty arrowheads indicate the L1, L2 and L3, respectively. MZ meristematic zone, TZ transition zone, bd boundary between differentially stained regions in R roots. Scale bar (**N**) referring to all images: 20 μm.

The results of Evans Blue staining show that in C, S and R roots (the last below the boundary) the dye is limited to the root surface and hardly ever penetrates the plasma membrane. Figure 5A–C,E clearly indicate that, in the mentioned cases, the stain does not enter the cytoplasm (except for single cells in the transition zone of the S roots, Figure 5D), but rather it associates with the two pellicle layers (L2 and L3) and the outer cell wall (L1) overlying young epidermal cells. In all groups (C, S and R) at 1 mm level (meristematic zone) all the three layers are distinguishable: the cell wall L1 directly surrounding protodermal cells, the outer pellicle layer L3 forming a wave-like pattern (especially distinct in S and R roots) and the inner layer of the pellicle L2 visible between L1 and L3 (Figure 7A–C). A similar staining pattern can be observed at the boundary in R roots, only L3 is less pronounced (Figure 7G). At 2 mm level (transition zone), L2 and L3 are indistinguishable, yet stained, and the L1 is clearly marked in all groups (Figure 7D–F).

To verify the presence of both pellicle layers L2 and L3, we used Coomassie blue stain, which was proved to be specific for the L3 layer [3]. The staining appears clear and intense in the meristematic zone, although in the stressed and recovering roots L3 is twice as thick as in the control roots (Figure 7H–J). In the transition zone of C and S roots the stain is rather pale, yet continuous (Figure 7K,L). Moreover, in S roots L3 is a bit thicker than in C roots. The staining pattern loses its continuous character at the boundary level of R roots (Figure 7N) and becomes thin and patchy at the 2 mm level (Figure 7M). The results of Evans Blue and Coomassie blue staining are summarized in Table 1 in Discussion.

#### 3.3.2. Changes in the Fibrillar Organization

**Figure 8:**
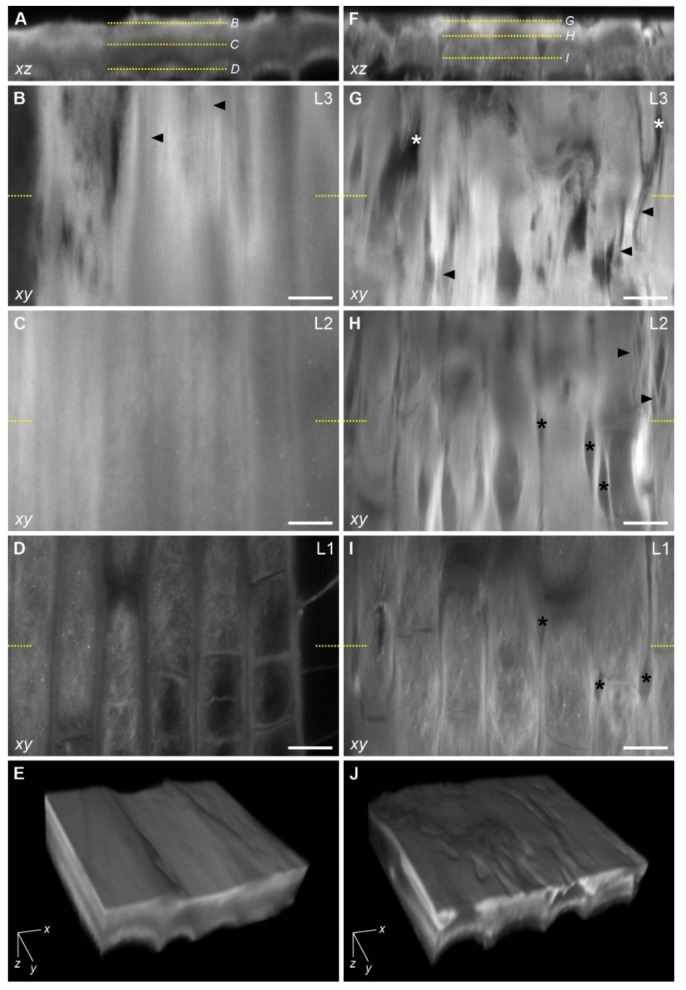
Confocal laser scanning microscope images of the epidermal surface of a representative control root (**A**–**E**) and stressed root (**F**–**J**). (**A**,**F**) Orthogonal projections (*xz*) of z-stacks; yellow dotted lines indicate levels of single optical sections (*xy*) through the L3, L2, L1 presented in (**B**–**D**) and (**G**–**I**). Arrowheads in (**B**,**G**,**H**) point to fibrillar structures, and asterisks in (**G**–**I**) show microgaps in the epidermal surface. Short yellow lines in (**B**–**D**) and (**G**–**I**) refer to the exact plane of the projections shown in (**A**) and (**F**), respectively. (**E**,**J**) 3D reconstructions of the L1, L2, L3 in C root (**E**) and S root (**J**) with clearly distinguishable features of the root surface: smooth in (**E**) and furrowed in (**J**). Scale bars: 10 μm.

For a more detailed look at the epidermal surface in the meristematic zone and to visualize the fibrillar organization of its layers, confocal laser scanning microscope (CLSM) imaging was applied to Calcofluor White-stained apices (Figure 8).

**Figure A1:**
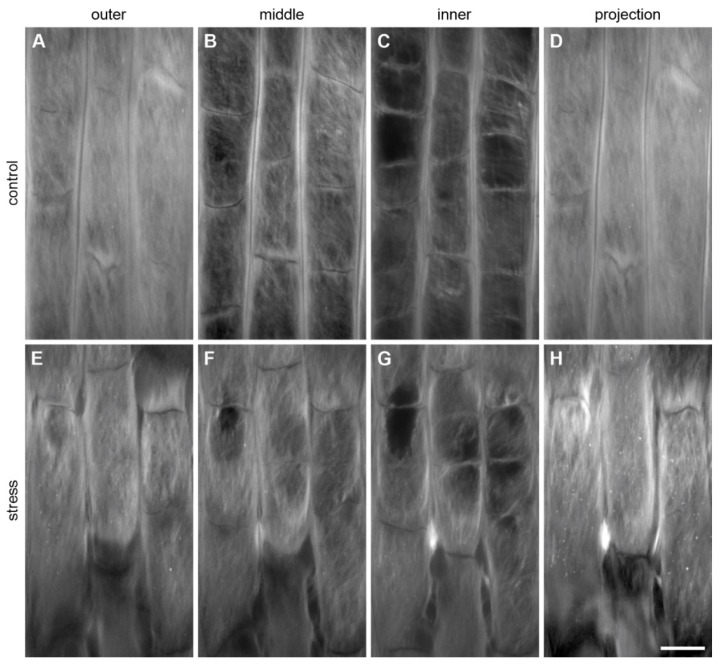
Orientation of cellulose fibrils in successive lamellas of the outer periclinal cell wall of the protoderm (L1) in control (**A**–**D**) and stressed (**E**–**H**) roots. The orientation of microfibrils changes from longitudinal or diagonal in the outermost lamella (**A**,**E**), through more oblique in the middle lamellas (**B**,**F**) to close to transverse in the innermost lamellas (**C**,**G**). (**D**,**H**) ImageJ maximum projection of z-stack through the L1. Scale bar referring to all images: 10 μm.

A transverse view (orthogonal projection of z-stack) of the epidermal surface is shown in Figure 8A,F in control and stressed roots, respectively. Longitudinal sections (individual xy slices from z-stack) taken from the levels referring to the three layers of the epidermal surface (indicated in Figure 8A,F) are presented in Figure 8B–D,G–I in control and stressed roots, respectively. In C roots, single longitudinally oriented fibrils are visible in the outermost layer, L3 (Figure 8B), while in L2 a fibrillar organization is difficult to resolve and the layer appears to be a homogeneous structure (Figure 8C). In the L1 (cell wall rounding out protodermal cells), cellulose fibrils are clearly distinguishable (Figure 8D). Detailed examination of successive optical sections through L1 shows its polylamellar construction, in which the orientation of microfibrils varies from lamella to lamella (Figure A1A–D in Appendix A). In stressed roots, both in L3 and L2, coarse longitudinally oriented slightly waving fibrils occur (Figure 8G,H). In the L1, a fine yet clearly visible meshwork of cellulose fibrils is present (Figure 8I). The changing orientation of the fibrils in successive L1 lamellas is shown in Figure A1E–H in Appendix A. In the epidermal surface of the stressed roots, significant damage of different depths is recognizable. The damage resembles gaps elongated along the root axis, in most roots penetrating all three layers (Figure 8G–I). In the L2 and L3 the gaps appear as tears in the fibrillar network (Figure 8G,H), while in the L1 they are visible as a local loss of junction between files of adjacent protodermal cells (Figure 8I).

In Figure 8E,J, three-dimensional visualization of the L1, L2 and L3 is shown in control roots and in stressed roots, respectively. In this view, the root surface (upper surface of the model) corresponds to the xy plane. In control roots (Figure 8E), natural furrows are visible, but in general, their surface is smooth, while in stressed roots (Figure 8J) the surface is irregular with numerous local indentations.

#### 3.3.3. Microdamage in the Root Superficial Layer

**Figure 9:**
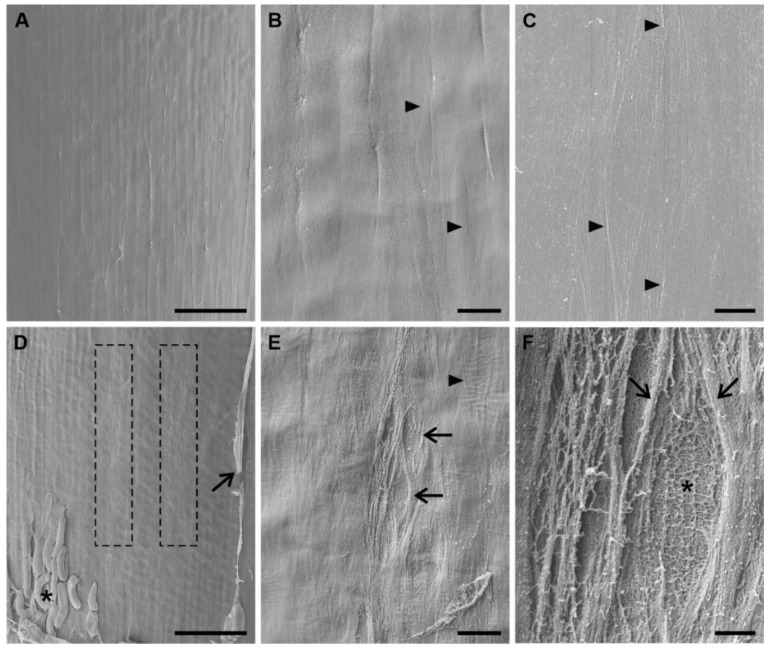
SEM micrographs of the epidermal surface of maize roots up to 1 mm from the root tip. (**A**–**C**) Control roots with smooth surface; arrowheads in (**B**,**C**) indicate fine regularly arranged fibrils. (**D**–**F**) Stressed roots with locally damaged, torn-like surface (**D**, frames). (**E**) Enlarged view of the left framed area in (**D**) with fusiform tears arranged along the root axis (arrows) and local transverse wrinkles (arrowhead). (**F**) Detailed view of the fibril arrangement around (arrows) and inside (asterisk) the tear. Asterisk and arrow in (**D**) indicate loose root cap cells and bands of mucilage, respectively. All image orientation is parallel to the root axis. Scale bars: 100 μm (**A**,**D**), 10 μm (**B**,**E**), 2 μm (**C**,**F**).

**Figure 10:**
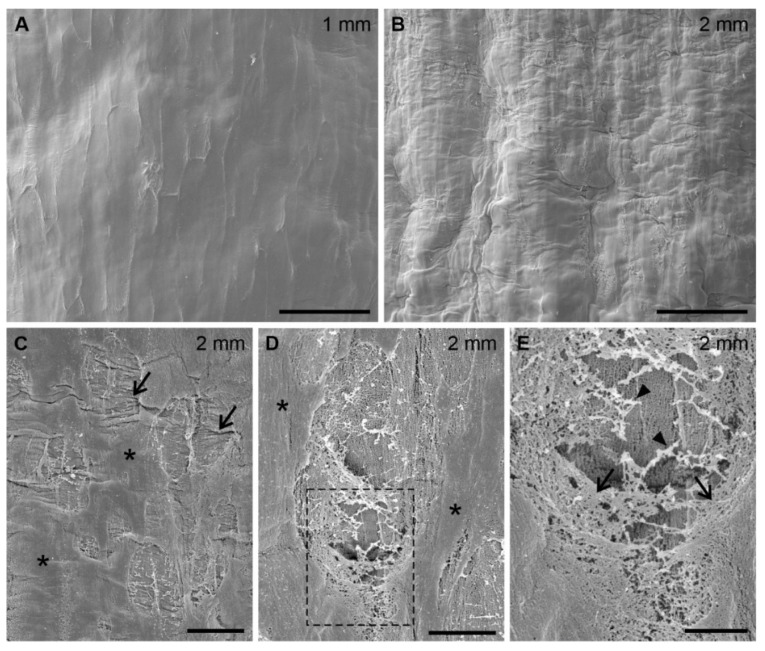
SEM micrographs of the epidermal surface of recovering maize roots below (**A**) and above (**B**) the boundary between the regions of living and dead cells (up to 1 mm and up to 2 mm from the root tip, respectively). (**C**,**D**) Subsequent enlargements of the root surface in (**B**). Arrows in (**C**) point to randomly distributed irregular damage and transverse microwrinkles, asterisks in (**C**,**D**) refer to areas of a compact structure occurring between the damage. (**E**) Further enlargement of the framed region in (**D**). Arrows and arrowheads indicate reticular network and thick fibrils, respectively. All image orientation is parallel to the root axis. Scale bars: 50 μm (**A**,**B**), 10 μm (**C**), 5 μm (**D**), 2 μm (**E**).

Results of microstructural analysis of the superficial root layer are presented in Figure 9 and Figure 10. In the meristematic zone (up to 1 mm from the root tip), the surface of control roots is smooth with fine outlines of cell complexes (Figure 9A) and only single thin fibrils are visible (Figure 9B,C). The surface of S roots is locally ‘frayed’ or ‘torn’ (Figure 9D) in this region. At higher magnification, the damage becomes visible as a loose network of fibrillar material with small fusiform tears arranged in longitudinal rows (Figure 9E). The tears are framed by coarser fibrils, while inside the tears the fibrils form a more regular reticular-like pattern (Figure 9F). Here and there on the root surface, small transversely aligned wrinkles are discernible (Figure 9E). In C roots, no root cap cells or mucilage are present (Figure 9A), while in S roots many loose root cap cells attached to the surface and bands of mucilage produced by single cap cells can be observed (Figure 9D). Similar microstructural changes in the root surface (e.g., regularly arranged tears and wrinkles) were observed in the transition zone (not shown).

In recovering roots a clear difference in surface microstructure between the regions of living and dead cells (see Figure 4C,D and Figure 6F) is observed. Below the boundary separating the two regions (Figure 10A, up to 1 mm from the root tip) the root surface is smooth, rather featureless, and only fine outlines of cell complexes can be seen, as in control roots. Above the boundary (up to 2 mm from the root tip) the texture of the surface is quite irregular and pleated (Figure 10B). At higher magnification, holes and damage with numerous transverse microfolds or wrinkles in the surface layer are visible (Figure 10C). Unlike in S roots (compare to Figure 9E,F), the damage is randomly distributed and of irregular shape. Enlarged views of a single hole and its neighborhood reveal the altering, non-uniform structure of the superficial layer, grading from compact and amorphous around the holes to reticulate and fibrillar at the edges of the hole with thicker fibrils within the hole (Figure 10D,E).

## 4. Discussion

The fine structure and development of the epidermal surface of grass roots has been described in detail by Abeysekera and McCully [3,4,6]. In this current paper, based on the existing information and using contemporary microscopic techniques, we attempt to further explore the character and the role of the epidermal surface. Our results (summarized in Table 1) address two main questions: (1) Does an external mechanical stress (similar to that present in natural environment) alter the structure of the epidermal surface? (2) Does the pellicle play a protective role for the root apex against mechanical stress?

### 4.1. On the Impact of Mechanical Stress on the Structure of the Epidermal Surface

External mechanical stress obviously changes the structure of the epidermal surface in maize roots. The changes mostly concern the pellicle itself, that is layers L2 and L3. Gaps and tears indicate serious damage on the surface of the roots tightly pressed into tubes (Figure 8 and Figure 9). The most extreme effects of the stress concern the disintegration of the L3 in the region of dead epidermal cells in roots that grew out of the tube (Figure 7 and Figure 10). Although the L1 (protodermal cell wall), which is the deepest layer of the epidermal surface, seems to be least affected, the CLSM images revealed damage also in this layer. However, despite this damage and significant stress applied (0.65 MPa, see [18]) the root tip cells hardly ever took up Evans Blue and/or propidium iodide, at least while the root was growing in the tube (Figure 5 and Figure 6).

In SEM micrographs the superficial layer resembles a locally torn fabric whose single threads separate, forming a hole. The damage probably results from friction against the internal wall of the plastic tube. In S roots the damage appears as tears elongated along the axis of the root apex that is parallel to the root growth direction (Figure 9). However, if we pressed a piece of fabric against some external surface and drew it along this surface (like a root rubbing against the inside of the tube) the tears would be probably elongated in a direction perpendicular to the movement. To try explaining this paradox we need to consider a cylindrical object subjected to mechanical stress. In such an object the stress in the transverse direction (perpendicular to the axis) is twice as large as the stress in the longitudinal direction (parallel to the axis). This results in forming longitudinal tears on the surface of the object. Considering the root apex as a cone-shaped object, its elongated form allows us to assume a distribution of mechanical stress similar to that occurring in cylindrical object. In R roots, tears are not regularly distributed, and they have different shapes. We can speculate that this is because they arise in a different way than damage in S roots, as will be discussed in the subsequent section.

### 4.2. On the Protective Role of the Pellicle

Plants have evolved different forms of protection of their root apices against mechanical stress. Mechanically stimulated roots undergo swelling and show enhanced cap cell sloughing and mucilage secretion [29,30]; see also [9]. Our results and the study by the McCully’s group [4,5,6] indicate that the pellicle may be another strategy of apex protection for a mechanically impeded root [31]. McCully and Canny [5] suggest that the pellicle increases the root tip stiffness, playing a role comparable to that of an aglet in a shoelace ending, protecting it from splitting and facilitating its passing through the eyelet hole. Such a coherence of the epidermal surface probably results from the L2 presence, because in maize mutants whose L2 is defective, either individual cells or cell packets form bulges sticking out over the epidermal surface [4]. However, our study shows that of the two pellicle layers, it is the L3 that seems to be especially protective. In the R roots where L3 is discontinuous and irregular, young epidermal cells become permeable to Evans Blue, which indicates cell death (Figure 5). Thus, based on the results of previous studies and those presented in this work, it can be concluded that both layers of the pellicle are needed to fully protect the root apex. The dark blue epidermal cells are clearly recognizable in the basal area of R apices, contrasting with the light blue region of living cells with untouched pellicle of which both layers L2 and L3 are distinguishable (Figure 7C,J). Single, dead epidermal cells also occurred in S roots approximately 2 mm above the root/cap junction. In this region, both the L2 and the L3 can be distinguished, but the L3 is unclear (Figure 7L) and torn (not shown), which may result in weaker protection against friction and, consequently, in the death of some cells. Thus, it can be assumed that epidermal cell death results from damage to the pellicle, caused by the mechanical stress created when the apex breaks through the tube. However, it is necessary to mention that the death of epidermal cells may be a more complex process regulated by ethylene [32], which is produced in response to various environmental stresses [33].

Although most of the R roots showed a large area of dead epidermal cells, growth of these roots continued (albeit, initially retarded), which suggests that the condition of the root epidermis is not crucial for growth itself. This conclusion seems in agreement with the observation about the rather passive role of the epidermis in root growth control, in opposition to the more significant role of the cortex cells in this process [34,35]. The root growth slowdown after removal of mechanical stress was observed by other authors in their studies on the effect of mechanical impedance on roots of different plant species [36,37,38].

The pellicle covers the meristematic zone of the root, which for at least two reasons needs special protection in order to remain undamaged, firstly, because all root cells originate from the meristem and, secondly, because it is only the apical part of the root that is responsible for active soil penetration [39]. It was proved that roots whose apices were covered with a defective pellicle were short-lived (after [4]). This shows how essential the role of the pellicle is in protecting the root apex.

Unfortunately, the effect of mechanical impedance on root epidermis has not previously been investigated and so it is difficult to compare our results to any other data. The impact of mechanical stress on the root surface was mostly explored in terms of the root cap response [40,41,42,43]. In monocotyledons (like maize) the root cap does not cover the entire meristematic zone but only its apical part while in dicotyledons, the whole meristem is covered by the lateral root cap [44,45]. This might be a possible reason for a special meristem protection, which is a complex and stress-resistant epidermal surface. Abeysekera and McCully [6] showed that the pellicle, together with the L1, was easy to remove from undisturbed root apices of maize, via gentle cutting and peeling. We tried the same method in the stressed roots. Unfortunately, it was not possible to isolate the pellicle in our experiments as it underwent tearing during attempts of removal. The possible reason might be the microdamage/microtears that made the epidermal surface discontinuous. As mentioned by Abeysekera and McCully [4], in the *Ageotropic* mutant, whose pellicle is defective, removing intact fragments of the epidermal surface is also impossible.

### 4.3. On the Boundary between the Regions of Living and Dead Cells

The boundary between dead and living cells is worth more consideration as its position and character are hard to interpret. To understand this difficulty, one should be aware of the specific characteristics of growth and cell production of the root (and of other plant organs). Such growth, named symplastic, is continuous, and neighboring cells preserve their contacts during their lifetimes [46]. In roots, new cells resulting from divisions in the meristem are continuously exiting the zone either towards the root cap or towards the root base, where they undergo elongation [47,48].

Taking into consideration the character of growth and relying on our results we may interpret the occurrence of the boundary in the following way. Below the boundary (region of living cells) there are existing meristematic cells that have left the tube undamaged due to strong protection from the root cap and due to a thick pellicle, and/or cells produced immediately after leaving the tube. The argument for these assumptions may be the presence of only living cells in the protoderm of short R roots (Figure 4E). Moreover, the pellicle is produced in the cone-shaped region of the root tip where the root circumference is smaller, under the layers of the root cap cells [3]. Thus, the newly formed protodermal cells are covered with a pellicle from the very beginning, and therefore, they do not experience friction due to the smaller size of the tip. Above the boundary (region of dead cells) the epidermis is probably damaged by friction of the root apex against the sharp tube edge. In the experiment with obliquely cut tube endings, the edge becomes even sharper, and this eventually results in a change of growth orientation to horizontal (Figure 4F). The root tip turns towards the shorter tube ending, which means that it grows slower on this side than on the opposite side. The boundary between the dead and living epidermal cells becomes parallel to the root axis due to the root reorientation. In light of the above conclusions, the boundary may be interpreted as a separation between the region of the ‘old’ meristem, whose protoderm has been damaged (dark area), and the ‘younger’ meristem, whose cells are produced after exiting the tube (pale area).

Here, we need to recognize two possible sources of friction experienced by the treated roots: one within the tube and a second at the tube ending. In the first case, the root surface rubs against the relatively large and smooth inner wall of the tube, but the friction is relieved by the root cap mucilage and sloughed root cap cells, which make damage of the pellicle slighter (like in S roots). In the second case, the root apex, whose external cells might have been damaged before by the friction against the tube wall, is exposed to rubbing against the sharp edge, which results in a complete lack of protection from the impaired pellicle and eventually in more severe and irregular damage (like in R roots). In our experiment, the two sources of friction imitate sharp-edged and smooth-edged obstacles naturally occurring in soil and in some cases affecting the root orientation [49,50].

To sum up, although the epidermal surface undergoes changes under mechanical stress (Table 1), only some of these changes appear to be adaptive. As we believe, damage and tears to the surface are simply the result of frictional forces. On the other hand, thickening of the L3 layer in the meristematic and transition zones may lead to increased protection of the root surface in the presence of stress. Both zones play a specific role in root growth and development: in the meristematic zone new cells are continuously generated, and the transition zone is the perception site for various environmental stimuli [51]. We can also speculate that epidermal cell death within the transition zone may be another element of the adaptive mechanism, because the dead cells may provide additional protection coating the deep tissues. However, this supposition requires verification by experiments.

### 4.4. On the Root Surface Imaging

As mentioned, the only known previous research on pellicle in maize is that from the McCully’s group, who applied light microscopy [7] and transmission electron microscopy [3] to observe its structure. Using confocal laser scanning microscopy and scanning electron microscopy enabled visualization of hitherto unobserved microdamage on the root surface. Each of the applied microscopic techniques shows different features and the combination of the two gives a completer and more comprehensive picture. Finally, it is worth mentioning that Evans Blue, which is a commonly used dye for cell death detection [52], to our knowledge, was used here for the first time to visualize the pellicle. Evans Blue was shown to be applicable in protein detection and its structure is similar to Coomassie brilliant blue R-250 [53]. As the latter is specific for the L3 [3], using Evans Blue in epidermal surface studies proved to be an apt choice.

## Figures and Tables

**Table 1:** Changes in the surface of mechanically treated maize roots.

	Control Root	Stressed Root	Recovering Root
	**Meristematic zone (MZ)**		
*Viability*	all protodermal cells alive	all protodermal cells alive	all protodermal cells alive
*Outer pellicle layer L3*	continuous	continuous, thicker compared to control	continuous, thicker compared to control
*Surface microstructure*	smooth and featureless	regularly arranged fusiform tears	smooth and featureless
	**Transition zone (TZ)**		
*Viability*	all young epidermal cells alive	single dead young epidermal cells	all young epidermal cells dead
*Outer pellicle layer L3*	continuous, less pronounced than in MZ	continuous, thicker compared to control, less pronounced than in MZ	discontinuous and diffuse
*Surface microstructure*	smooth and featureless	regularly arranged fusiform tears	wrinkles, irregular damage/holes

## Data Availability

Not applicable.

## Appendix B. Application Details of the Used Staining Techniques

**Table A1:** Specification of the staining techniques.

Stain	Specificity/Detection	References
Evans Blue	Cell viability	[52]
	Proteins	[53]
Propidium iodide	Cell viability/DNA (in dead cells)	[54,55]
	Homogalacturonan	[56]
Coomassie brilliant blue R-250	Proteins	[57]
Calcofluor White	β-glucans (cellulose, xyloglucan, callose), chitin	[58,59]
